# Supplementary material for: An Integrated Research–Clinical BSL-2 Platform for a Live SARS-CoV-2 Neutralization Assay
Source: Viruses. 2023 Aug 31;15(9):1855. doi: 10.3390/v15091855 (PMC10536566; doi:10.3390/v15091855)
Supplement: Supplementary file 1 [file viruses-15-01855-s001.zip › viruses-2583514-supplementary.pdf]

Supplementary Materials

Table S1. $FRNT_{50}$s of 30 human serum/plasma specimens collected before COVID-19 emergence.

Sample ID	WA1		BA.5		XBB.1.5	
	$^{\$}PRNT_{50}$	$^{\#}FRNT_{50}$	$PRNT_{50}$	$FRNT_{50}$	$PRNT_{50}$	$FRNT_{50}$
N1	<20	<20	<20	<20	<20	<20
N2	<20	<20	<20	<20	<20	<20
N3	<20	<20	<20	<20	<20	<20
N4	<20	<20	<20	<20	<20	<20
N5	<20	<20	<20	<20	<20	<20
N6	<20	<20	<20	<20	<20	<20
N7	<20	<20	<20	<20	<20	<20
N8	<20	<20	<20	<20	<20	<20
N9	<20	<20	<20	<20	<20	<20
N10	<20	<20	<20	<20	<20	<20
N11	<20	<20	<20	<20	<20	<20
N12	<20	<20	<20	<20	<20	<20
N13	<20	<20	<20	<20	<20	<20
N14	<20	<20	<20	<20	<20	<20
N15	<20	<20	<20	<20	<20	<20
N16	<20	<20	<20	<20	<20	<20
N17	<20	<20	<20	<20	<20	<20
N18	<20	<20	<20	<20	<20	<20
N19	<20	<20	<20	<20	<20	<20
N20	<20	<20	<20	<20	<20	<20
N21	<20	<20	<20	<20	<20	<20
N22	<20	<20	<20	<20	<20	<20
N23	<20	<20	<20	<20	<20	<20
N24	<20	<20	<20	<20	<20	<20
N25	<20	<20	<20	<20	<20	<20
N26	<20	<20	<20	<20	<20	<20
N27	<20	<20	<20	<20	<20	<20
N28	<20	<20	<20	<20	<20	<20
N29	<20	<20	<20	<20	<20	<20
N30	<20	<20	<20	<20	<20	<20

$^{\$}$PRNT was performed in duplication using infectious clone-derived SARS-CoV-2 containing wild-type (WA1) or variants' spike on VeroE6 cells. The geometric means of $PRNT_{50}$ from the duplicates are shown.

$^{\#}$FRNT was performed in duplication using live-attenuated mGFP Δ3678 SARS-CoV-2 containing wild-type (WA1) or variants' spike on A549-hACE2 cells. The geometric means of $FRNT_{50}$ from the duplicates are shown.

Table S2. $FRNT_{50}$ and $PRNT_{50}$ values of 20 human serum/plasma specimens collected about one month after 2 doses of Pfizer-BioNTech COVID-19 mRNA vaccination.

Sample ID	$^{\$}PRNT_{50}$ (WA1 SARS-CoV-2, VeroE6)	$^{*}FFRNT_{50}$ (mGFP Δ3678, VeroE6)	$^{\#}FRNT_{50}$ (mGFP Δ3678, A549-hACE2)
1	113	84	863
2	320	177	2722
3	320	296	1260
4	320	257	292
5	381	351	2472
6	381	135	1134
7	453	359	1044
8	453	243	1107
9	453	588	2439
10	538	356	1981
11	538	311	2704
12	640	458	2787
13	640	493	4815
14	640	485	725
15	640	443	1055
16	640	318	1310
P-17	905	622	2217
P-18	905	804	2101
P-19	905	703	1923
P-20	1280	793	2598

$^{\$}$PRNT was performed twice in duplication using infectious clone-derived SARS-CoV-2 containing wild-type (WA1) spike on VeroE6 cells. The geometric means of $PRNT_{50}$ from all replicates are shown.
*FFRNT was performed in duplication using live-attenuated mGFP Δ3678 SARS-CoV-2 containing wild-type (WA1) spike on VeroE6 cells. The geometric means of $FFRNT_{50}$ from the duplicates are shown.
$^{\#}$FRNT was performed in duplication using live-attenuated mGFP Δ3678 SARS-CoV-2 containing wild-type (WA1) spike on A549-hACE2 cells. The geometric means of $FRNT_{50}$ from the duplicates are shown.

Table S3. $FRNT_{50}$ and $PRNT_{50}$ values of 30 human serum/plasma samples collected from individuals with BA.5 infection and/or bivalent booster.

Sample ID	#$FRNT_{50}$										$$PRNT_{50}$			*$FRNT_{50}/PRNT_{50}$
	Test 1		Test 2		Test 3		Test 4		GMT	%CV				
	Analyst 1	Analyst 2	Analyst 1	Analyst 2	Analyst 1	Analyst 2	Analyst 1	Analyst 2			PRNT-1	PRNT-2	GMT	
1	30	32	34	37	49	54	44	47	40	21.58	20	40	28	1.42
2	107	85	112	98	160	146	126	129	118	20.67	40	40	40	2.96
3	255	181	246	188	300	308	271	211	241	19.68	80	40	57	4.26
4	112	81	93	94	153	147	114	123	112	22.42	40	40	40	2.80
5	561	368	442	327	534	391	535	446	443	19.13	80	40	57	7.83
6	551	430	1140	903	700	767	639	658	696	30.26	320	160	226	3.07
7	504	474	514	875	742	762	717	700	646	22.03	160	160	160	4.04
8	358	377	518	580	483	438	421	420	444	16.46	160	160	160	2.78
9	622	778	732	1069	934	839	960	773	827	17.06	160	320	226	3.66
10	470	485	940	756	718	637	604	600	636	23.53	160	160	160	3.98
11	577	586	972	782	856	768	842	757	757	17.39	160	320	226	3.34
12	623	667	1340	937	1106	937	916	900	904	24.55	320	320	320	2.82
13	1604	1099		1510	1421	1507	1540	1295	1415	12.23	640	640	640	2.21
14	1401	1301	2730	2555	1994	1736	1875	1749	1862	26.31	640	320	453	4.11
15	1413	1274	2357	1974	2162	2065	1969	1879	1852	19.45	640	640	640	2.89
16	619	587	1293	870	1052	801	841	791	832	26.67	320	640	453	1.84
17	1954	1927	2130	1908	2844	3095	2346	2363	2286	19.08	2560	640	1280	1.79
18	1729	1681	1668	1438	2189	2225	2065	1801	1831	15.16	1280	640	905	2.02
19	4530	4880	6006	4470	7108	8095	6018	6513	5832	21.64	2560	5120	3620	1.61
20	3074	3248	3146	2946	4327	3766	3385	3274	3372	13.21	2560	1280	1810	1.86
21	3116	3262	3272	3464	5059	4535	4242	3920	3805	18.21	1280	2560	1810	2.10
22	6422	5988	5812	5292	7183	6017	6579	5912	6128	9.27	5120	5120	5120	1.20
23	5838	4776	4114	5202	7850	7025	6471	6770	5886	20.88	2560	2560	2560	2.30
24	7936	6386	7922	6062	9269	8216	7598	6974	7482	13.80	5120	2560	3620	2.07
25	10488	8822	9026	6718	8401	6903	7797	9196	8337	14.89	2560	5120	3620	2.30
26	12305	13735	17392	13970	16480	15088	12230	21215	15062	19.67	10240	20480	14482	1.04
27	7500	7700	11090	11116	10078	11060	8183	11260	9616	17.12	5120	5120	5120	1.88
28	8330	8620	11714	9044	10903	10933	10560	10340	9987	12.26	2560	5120	3620	2.76
29	28990	33905	41700	38120	37578	38970	27885	34373	34884	13.80	20480	20480	20480	1.70
30	9045	7405	8438	8774	9100	9673	9515	11703	9138	13.37	5120	5120	5120	1.78

$PRNT was performed twice in duplication using infectious clone-derived SARS-CoV-2 containing BA.5 spike on VeroE6 cells.

#FRNT was performed in duplication using live-attenuated mGFP Δ3678 SARS-CoV-2 containing BA.5 spike on A549-hACE2 cells.

*Ratios were calculated by dividing the geometric means of $FRNT_{50}$ to the geometric means of $PRNT_{50}$.

GMT: geometric mean titer.

CV: coefficient of variation.

Table S4. $FRNT_{50}$ and $PRNT_{50}$ of 25 human serum/plasma collected from individuals after XBB.1.5 infection.

Sample ID	#$FRNT_{50}$ Test 1	Test 2	Test 3	GMT	$PRNT_{50}$ $PRNT_{50}$-1	$PRNT_{50}$-2	GMT	%CV	*$FRNT_{50}/PRNT_{50}$
1	420	415	546	457	160	160	160	16.02%	2.85
2	1724	1348	1596	1548	640	640	640	12.29%	2.42
3	156	120	225	162	80	80	80	31.77%	2.02
4	323	223	392	304	80	80	80	27.19%	3.80
5	279	232	424	301	80	80	80	32.18%	3.77
6	188	144	249	189	80	80	80	27.26%	2.36
7	1138	1004	1533	1205	160	320	226	22.45%	5.33
8	53	37	53	47	40	40	40	19.16%	1.18
9	297	420	251	315	80	80	80	27.21%	3.94
10	647	1077	1284	963	320	320	320	32.43%	3.01
11	979	897	885	919	160	160	160	5.55%	5.74
12	501	605	586	562	80	80	80	9.80%	7.03
13	433	560	484	490	80	160	113	13.06%	4.33
14	30	27	35	31	40	40	40	13.45%	0.76
15	634	378	414	463	80	80	80	29.15%	5.78
16	670	485	512	550	160	160	160	18.01%	3.44
17	104	79	120	100	80	80	80	20.31%	1.25
18	1449	782	639	898	160	320	226	*45.20%*	3.97
19	256	246	324	273	80	80	80	15.35%	3.41
20	101	74	123	97	40	40	40	24.81%	2.43
21	723	672	951	773	80	80	80	18.98%	9.67
22	546	508	593	548	80	80	80	7.73%	6.85
23	320	234	223	256	80	80	80	20.44%	3.20
24	152	92	162	132	40	40	40	28.02%	3.29
25	223	156	227	199	40	80	57	19.59%	3.50

$PRNT was performed using infectious clone-derived SARS-CoV-2 containing XBB.1.5 spike on VeroE6 cells.

#FRNT was performed using live-attenuated mGFP Δ3678 SARS-CoV-2 containing XBB.1.5 spike on A549-hACE2 cells.

*Ratios were calculated by dividing the geometric means of $FRNT_{50}$ to the geometric means of $PRNT_{50}$.

GMT: geometric mean titer.

CV: coefficient of variation.

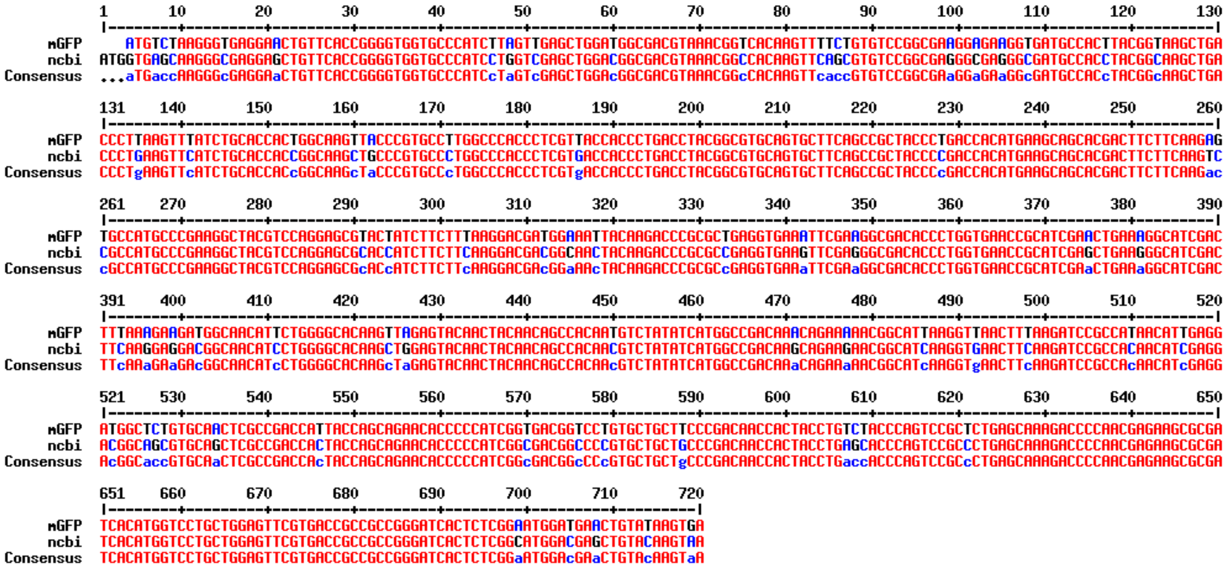

Figure S1. Sequence alignment. A web server (http://multalin.toulouse.inra.fr/multalin/) was used to align the nucleotide sequence of mGFP and a reference GFP obtained from NCBI (GenBank accession code, MH325108.1). Identical sequences are shown in red. Miss-matched nucleotides are shown in blue and black.

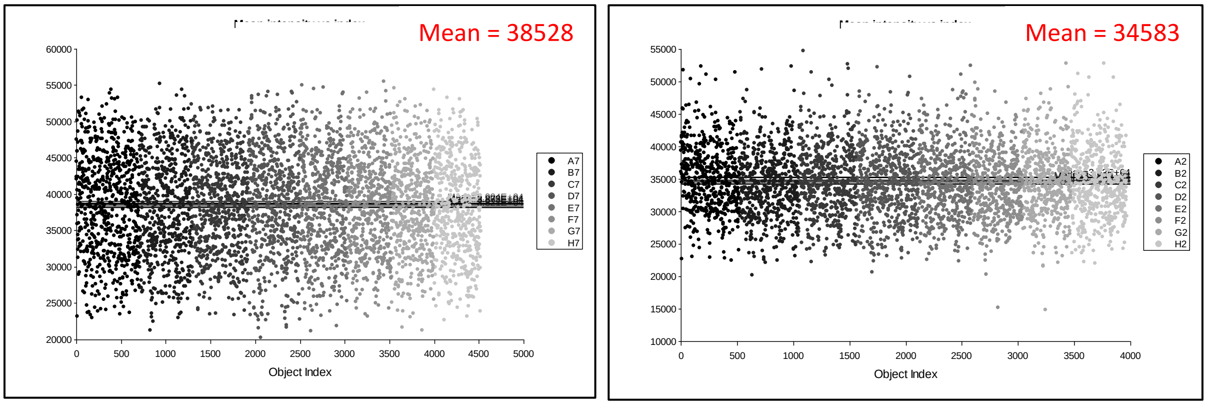

Figure S2. Fluorescence intensity of mGFP (left) and mNG (right) Δ3678 foci. Each dot represents one green fluorescent focus acquired by Cytation 7 imager. Numbers in red indicate the mean green fluorescence intensity of the foci.

A

BA.5-spike (EPI_ISL_11542604**)**

T19I, del24-26, A27S, G142D, V213G, G339D, S371F, S373P, S375F, T376A, D405N, R408S, K417N, N440K, L452Q, S477N, T478K, E484A, Q493R, Q498R, N501Y, Y505H, D614G, H655Y, N679K, P681H, S704L, N764K, D796Y, Q954H, N969K

XBB.1.5-spike (EPI_ISL_16292655)

T19I, del24-26, A27S, V83A, G142D, del144, H146Q, Q183E, V213E, G252V, G339H, R346T, L368I, S371F, S375F,T376A, D405N, R408S, K417N, N440K, V445P, G446S, N460K, S477N, T478K, E484A, F486P, F490S, Q498R, N501Y, Y505H, D614G, H655Y, N679K, P681H, N764K, D796Y, Q954H, N969K

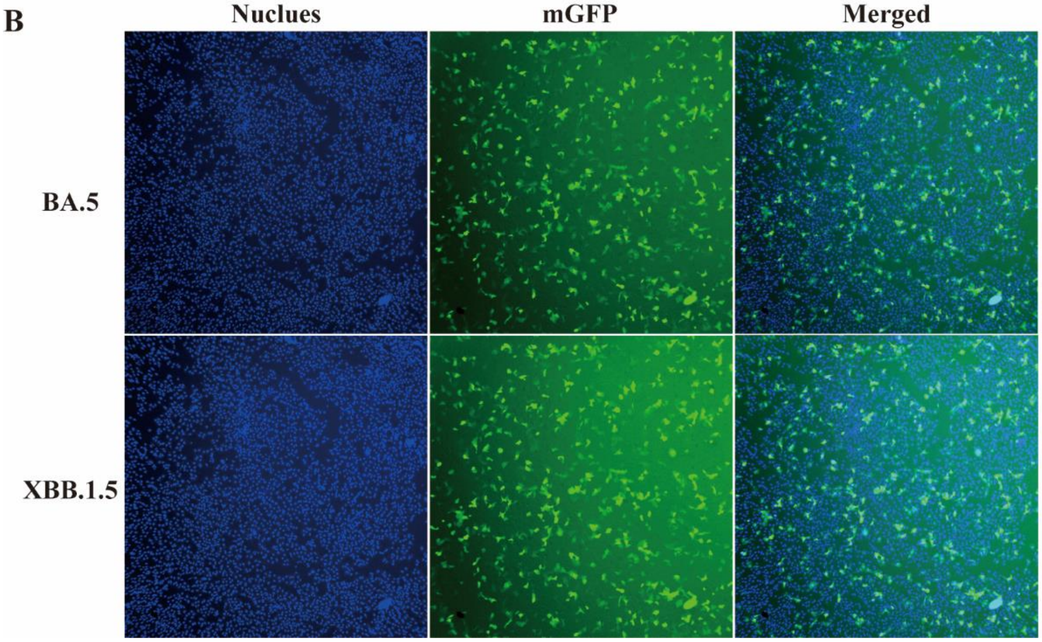

Figure S3. Construction of mGFP Δ3678_BA.5-spike and mGFP Δ3678_XBB.1.5-spike. (A) Mutations in BA.5-spike and XBB.1.5-spike compared to the parental strain USA-WA1/2020. (B) Representative images of A549-hACE2 cells infected by mGFP Δ3678_BA.5-spike (top) and mGFP Δ3678_XBB.1.5-spike (bottom) at 16 h post-infection.
